# Dynamic evaluation of a vapor compression refrigeration cycle combined with a PCM storage tank for improving condenser performance

**DOI:** 10.1016/j.heliyon.2024.e35837

**Published:** 2024-08-06

**Authors:** Alireza Riahi, Mohammad Behshad Shafii

**Affiliations:** a Department of Mechanical Engineering, Sharif University of Technology, Tehran, Iran; b Sharif Energy, Water and Environment Institute (SEWEI), Tehran, Iran

**Keywords:** *Vapor compression cycle*, *PCM*, *Peak load shaving*

## Abstract

In this study, a vapor compression refrigeration cycle integrated with a phase change material (PCM) storage tank has been dynamically simulated over a 24-h period. The primary objective of this system is to reduce electric energy consumption during on-peak hours (12:00–19:00) and shift it to off-peak hours (1:00–10:00). During off-peak hours, the vapor compression refrigeration system stores cooling energy in the PCM storage tank. The stored cooling energy is then reused during on-peak hours to pre-cool the condenser inlet refrigerant, enhancing the cooling system's performance and reducing electric consumption during on-peak hours. Oleic acid, eutectic mixtures of 45 % capric acid and 55 % lauric acid by weight (CL), a commercial blend of salt hydrate and paraffin wax (SP224A), and CaCl_2_.6H_2_O have been selected as the PCM mediums. The effects of weather conditions and PCM storage tank parameters on the system's performance have been investigated. The results indicate that SP224A performs the best in most weather conditions. The maximum electric peak load shaving occurred under Ramsar city weather conditions, achieving 98.85 %. Additionally, the compressor's electric energy consumption is reduced by about 23.38 %. Moreover, increasing the length of PCM storage pipes leads to a reduction in compressor energy consumption during on-peak hours, as well as electric peak load shaving, with an improvement of up to 156 %.

## 1 Introduction

Due to the growing global population and improving living conditions, there has been a substantial increase in energy consumption [1]. Approximately 40 % of the world's energy production is dedicated to meeting the energy needs of buildings, with a significant proportion utilized to ensure thermal comfort within these structures [2].

According to reports from the International Energy Agency (IEA), electricity consumption for cooling purposes in buildings accounted for 8.5 % in 2019. On average, global electricity consumption for cooling applications rises to approximately 15 % during on-peak hours. However, on the hottest days, residential cooling consumption can surge up to 50 %. This substantial increase in electricity demand during on-peak hours for cooling purposes poses the risk of blackouts and puts pressure on electricity transmission systems [3,4]. One potential solution to address this issue is to decrease electric energy consumption during on-peak hours by shifting it to off-peak hours. This can be achieved by storing cooling energy in a storage tank during periods of low demand and utilizing it during high-demand periods, such as on-peak hours [5,6]. Energy storage units are generally categorized as sensible, latent, and thermochemical [7]. Latent energy storage units are commonly preferred in home applications due to their lower complexity compared to thermochemical storage units and higher energy density compared to sensible storage units [8,9].

Yamaha and Misaki [10] conducted a study on the impact of electric peak load shifting in an office building in Japan. In their proposed approach, a phase change material (PCM) storage tank is charged from 5 to 8 using a cooling system, and then discharged from 13 to 16. The findings reveal that utilizing a 400 kg PCM with a melting temperature of 19 °C can meet the cooling requirements of a 73.8 m^2^ building during hot summer days. Gholami and Farid [11] conducted an experimental evaluation of a PCM storage tank in a building for heating and cooling purposes during both warm and cold seasons. The utilization of the PCM storage tank for cooling applications resulted in energy savings of approximately 30 % between March and April, considering the weather conditions in New Zealand. Additionally, they compared the performance of using PCM in passive applications (in building walls) versus active applications (in the storage unit). The findings revealed that the active system outperformed the passive system, achieving a 22 % reduction in electric energy consumption and 32 % reduction in electricity costs compared to the passive system [12]. Comodi et al. [13] investigated the thermal and economic efficiencies of employing a sensible cooling energy storage unit, considering the economic and weather conditions of Singapore. The study indicated that the payback period of the proposed system varied between 8.9 and 16 years, depending on different scenarios and storage tank volumes ranging from 482 to 2214 m^3^. Ruddell et al. [14] assessed the effectiveness of employing a cooling storage tank in a city within the USA. The results demonstrated that the implementation of the proposed system resulted in a 15 % decrease in electric energy consumption during on-peak hours. Additionally, it was found to reduce daily electricity bills by up to 2.47 $. Erdemir and Dincer [15] examined the impact of utilizing heating and cooling energy storage to shift the electric load from on-peak hours to off-peak hours, considering the weather and economic conditions of Canada. The study demonstrated that the use of energy storage for cooling applications could potentially reduce electric energy consumption by up to 45 % during on-peak hours. Additionally, it resulted in a reduction in cooling costs of up to 20 %. Furthermore, Riahi et al. [16] examined the effect of adding a PCM storage tank to an air conditioning system experimentally. The results indicated that the addition of a PCM storage tank can boost the COP of the system during on-peak hours by up to 86.34 %.

An alternative method to reduce electric peak load is by improving the performance of vapor compression refrigeration systems (VCRS) during on-peak hours. One approach is to pre-cool the refrigerant at the inlet of the VCRS condenser, which results in a reduction in condenser temperature. As a result, the condenser pressure decreases, leading to a reduction in the electric energy consumption of the compressor [17]. In this regard, Wang et al. [18] conducted an evaluation of the impact of incorporating a PCM storage unit at different locations within the VCRS for pre-cooling purposes. The study revealed that utilizing PCM-stored cooling energy at the condenser outlet stream to reduce the sub-cooled temperature could result in energy savings of up to 8 %. Furthermore, using PCM at the condenser inlet stream showed an improvement in the coefficient of performance (COP) by up to 6 %. Additionally, employing PCM at the evaporator outlet stream reduced the superheat temperature entering the compressor, leading to an enhanced COP by up to 7 % [19]. Waly et al. [20] assessed the performance of a VCRS in which the condenser's inlet air was pre-cooled, taking into account the weather conditions in Kuwait. The study found that implementing different cooling strategies could improve the COP by approximately 36 %–59 %. In this regard, Ibrahim et al. [21] claimed that a VCRS COP was improved by about 21.4 % by reducing condenser inlet air temperature up to 4 °C. Riahi et al. [22] investigated the impact of pre-cooling the condenser's inlet refrigerant stream in a VCRS. The study demonstrated that by storing cooling energy in a PCM storage tank and utilizing it during on-peak hours, the electric peak load could be reduced by approximately 76.3 % according to the weather conditions of Tehran city.

This study, building upon the research in Reference [22], examines the impact of pre-cooling the condenser's inlet refrigerant stream on the system's performance. Previous literature primarily focused on improving vapor compression cycle performance by modifying components such as the compressor, condenser, expansion valve, and refrigerant type. Only a few studies explored the use of PCM in refrigeration cycles to enhance performance and reduce electric peak load. Additionally, prior studies mainly concentrated on the charging process of PCM, neglecting the discharging process. Furthermore, the effects of key parameters and dynamic evaluation of the system are not thoroughly investigated. In this study, a dynamic assessment of a vapor compression refrigeration system combined with a PCM storage tank has been conducted using a 1-min time step over a 24-h period. The study also examined the influence of various weather conditions based on Ahvaz, Tehran, Ramsar, Hamadan, and Bushehr cities on the system's performance. Moreover, the study investigated the effects of PCM storage tank physical parameters, such as the number and length of pipes.

## 2 System description

**Fig. 1:**
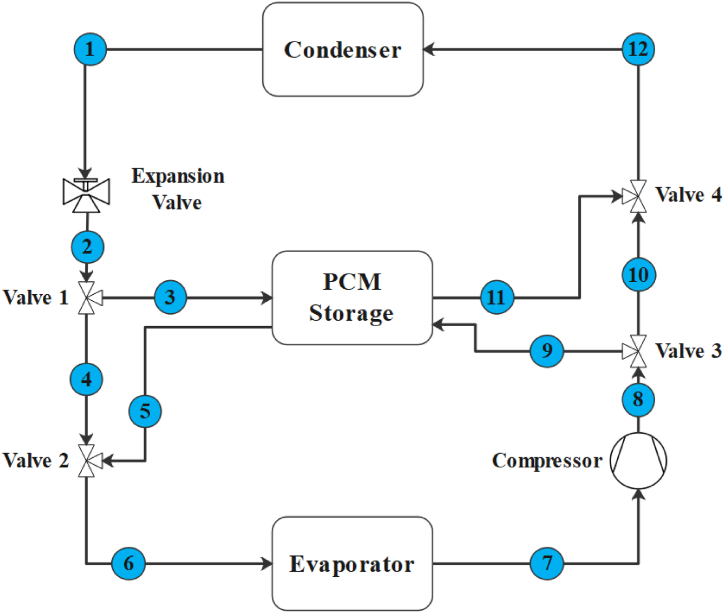
The Schematic diagram of the VCRS combined with a PCM storage tank for condenser pre-cooling during the charging and discharging processes.

As mentioned before, this study is the continuation of the survey in Ref. [22], with its schematic diagram shown in Fig. 1. During the charging process (storing cold energy in the PCM by solidifying), the cooling load produced by the VCRS is directed to the PCM storage tank (Point 3) from 1:00 to 10:00. Subsequently, the refrigerant, R134a in this study, enters the evaporator (Points 5 and 6) to provide the building cooling load. It then proceeds to the compressor and condenser to complete the typical refrigeration cycle.

During the discharge process (releasing cold energy from PCM by melting), coinciding with on-peak hours (12:00–19:00), the compressor outlet stream is directed to the PCM storage tank (Points 8 and 9). It is then cooled by the stored cooling energy in the PCM storage tank, resulting in a decrease in the condenser inlet stream temperature. Consequently, the condenser pressure drops, leading to a reduction in compressor power consumption. The condenser outlet stream (Point 1) then proceeds to the expansion valve, evaporator, and compressor (2 → 4 → 6 → 7) to complete the typical refrigeration cycle.

At other times of the day, the system functions like a conventional cooling system. The refrigerant outlet from the evaporator goes to the compressor (Point 7). After increasing its pressure, it enters the condenser (Point 12), then proceeds to the expansion valve (point 1). Finally, the cooled refrigerant goes back to the evaporator (Point 6) to absorb heat from the building.

## 3 System modeling

As this study is the continuation of the survey in Ref. [22] and no changes have been made in its modeling principles, the equations and validation are not presented. Instead, only a brief description of assumptions and modeling is provided as follows.

### 3.1 Heat exchangers

The heat exchangers used in this study include a condenser and an evaporator. The modeling is based on the effectiveness-NTU (number of transfer unit) model, considering both single-phase and two-phase conditions. The rate of transfer heat (U-values) of the heat exchangers are determined based on the geometry of common condensers and evaporators. The condenser outlet stream is assumed to be liquid. Pressure and heat losses in the heat exchanger are neglected. The geometric specifications of the condenser and evaporator used in this study are shown in Table 1.

**Table 1:** The specifications of the heat exchangers used in this study.

Component	Fan Blow Rate (L/s)	Tube Length (m)	Tube Diameter (m)	Number of Fin (#/in)
Condenser	1000	20	0.015	8
Evaporator	700	12	0.008	8

### 3.2 Compressor

In accordance with existing vapor compression refrigeration systems, a rotary-screw compressor with 4000 rpm and a volumetric inlet flow of 0.0495 L has been modeled. The modeling accounts for the isentropic, volumetric, and mechanical efficiencies of the compressor, while neglecting heat loss in the compressor [23]. The equations used to model the compressor are detailed in Table 2.

**Table 2:** The equations used to model the compressor.

Parameter	Equation
Temperature Ratio in Compressor	$\frac{T_{6}}{T_{5}}={\left(\frac{P_{cond}}{P_{evap}}\right)}^{\frac{\gamma -1}{\gamma }}$
The Refrigerant Mass Flow Rate	${\dot{m}}_{ref}=\eta_{v}\times \rho_{1}\times V_{G}\times \omega$
Volumetric Efficiency	$\eta_{v}=0.9207-0.0756\;\left(\frac{P_{6}}{P_{5}}\right)+0.0018{\left(\frac{P_{6}}{P_{5}}\right)}^{2}$
Compressor Power Consumption	${\dot{W}}_{comp}=\frac{{\dot{m}}_{ref}\left(h_{6,isen}-h_{5}\right)}{\eta_{total}}$
Compressor Total Efficiency	$\eta_{total}=\eta_{is}\eta_{mech}\eta_{elec}$
Compressor Isentropic Efficiency	$\eta_{is}=\begin{array}{c} 0.85-0.046667 \end{array}\;\left(\frac{P_{6}}{P_{5}}\right)$
Coefficient of Performance	$COP=\frac{{\dot{Q}}_{evap}}{{\dot{W}}_{comp}}$
Peak Load	${\dot{W}}_{maximum,comp}-\frac{\sum {\dot{W}}_{comp,conventional\;system}}{Operation\;Time}$
Peak Load Shaving	$\left(\;\frac{{Peak\;load}_{conventional\;system}-\;{Peak\;load}_{\;PCM\;integrated\;system}}{{Peak\;load}_{conventional\;system}}\right)\times 100$

### 3.3 PCM storage tank

The 150-L PCM storage tank consists of 20 horizontal pipes, each with a length of 1 m. Refrigerant flows inside the pipes, exchanging heat with the PCM, which covers the outside of the pipes. Heat loss from the PCM storage tank to the ambient has been considered negligible. The thermophysical specifications of the refrigerant and PCM are assumed to be constant. The thermodynamic properties of the PCM used in this study are listed in Table 3. The PCM has been chosen based on its application for space cooling. Moreover, for a proper latent heat transfer between PCM and refrigerant in the peak and off-peak loads, the melting point temperature of the PCM should be higher and lower than refrigerant after the expansion valve and compressor, respectively. This issue has been considered carefully for the selection of the specific PCM. Additionally, the outlet stream temperature of the expansion valve has been assumed to be 7°. The geometric specifications of the PCM storage tank used in this study are detailed in Table 4.

**Table 3:** The thermodynamic properties of the PCM used in this study [[24], [25], [26], [27]].

Properties	Oleic acid [26,27]	SP224A [25]	CL [25]	CaCl_2_.6H_2_O [24,25]
Melting Temperature (°C)	16	21.6	20.6	29.6
Latent Heat (kJ/kg)	207	125	188	212
specific heat in sold phase (kJ/kg. K)	1.74	1.7	1.8	1.77
specific heat in liquid phase (kJ/kg. K)	2.37	1.8	2.12	2.2
Heat Transfer Value at Solid Phase (W/m. °C)	0.139	0.6	0.14	1.08
Heat Transfer Value at Liquid Phase (W/m. °C)	0.12	0.4	0.13	0.56
Density at Solid Phase (kg/m^3^)	871	1490	890	1710
Density at Liquid Phase (kg/m^3^)	845	1440	770	1560

**Table 4:** The geometric specification of the PCM storage tank.

Item	Value
Number of Tubes	5–30
Length of Tubes (m)	0.5–2
Tube Wall Conductivity (W/m. K)	111
Tube Inner Diameter (m)	0.012
Tube Outer Diameter (m)	0.014

**Fig. 2:**
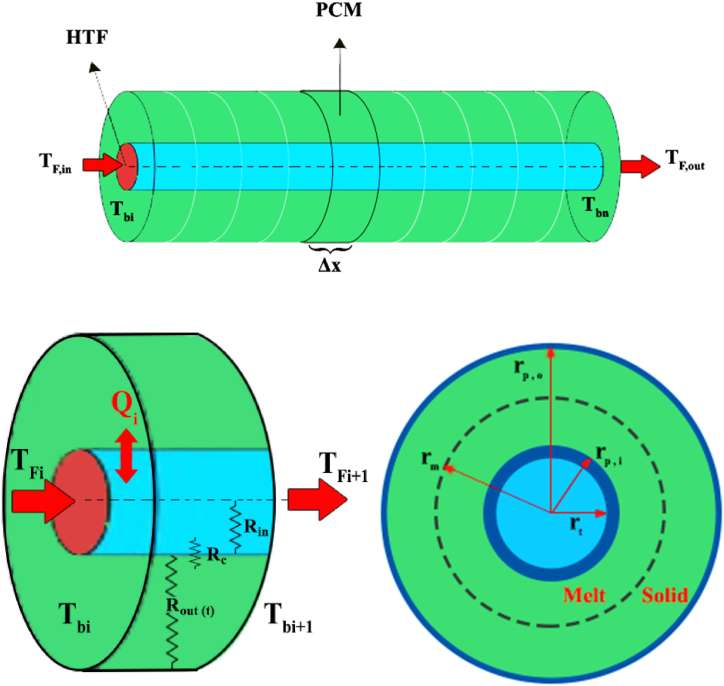
The schematic of differential control volume in the PCM heat exchanger (Reprinted with permission from Elsevier) [17].

To calculate the local heat transfer of PCM, a two-dimensional differential method is employed, predicting the amount of melting or solidification at any time and point. A schematic of the PCM heat exchanger control volume is illustrated in Fig. 2. Comparing the melting fraction of PCM modeled in this study with Ref [28] using its input data, the average relative error between the results of the simulated model and the model in Ref. [28] is 6 %.

### 3.4 Software simulation

**Fig. 3:**
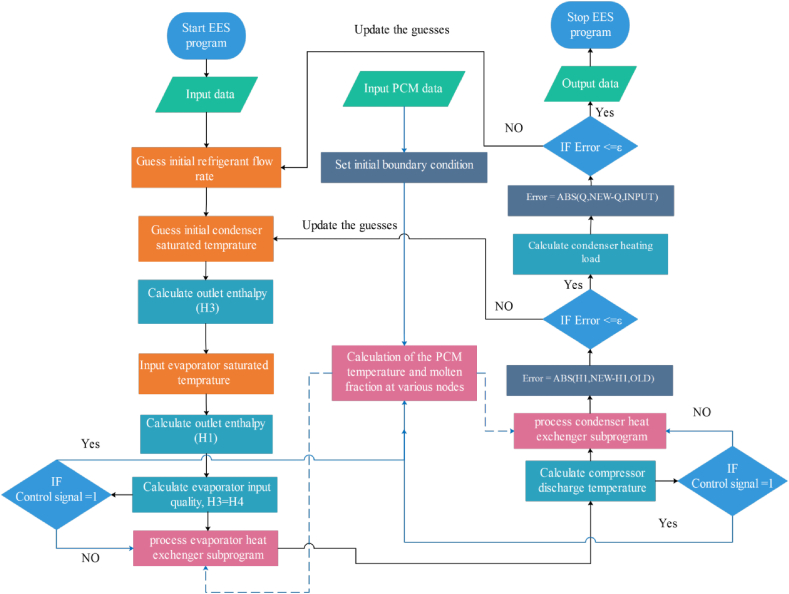
The Flow chart of the system modeled in EES software (Reprinted with permission from Elsevier) [17].

The proposed system has been dynamically modeled in the Engineer Equation Solver (EES) software over a day with a 1-min time step. The times for the charging (storing cold energy in the PCM by solidifying) and discharging (releasing cold energy from PCM by melting) processes have been set from 1:00 to 10:00 and 12:00 to 19:00, respectively. Table 5 provides the required input and output information to run the program. Additionally, the flow chart of the calculation steps and the general algorithm of the program in several iterative loops are illustrated in Fig. 3.

**Table 5:** The input and output of the program simulated in EES.

Inputs	Outputs
• The ambient temperature at any moment.	• Compressor power in normal cooling system.
• The temperature of the space in the evaporator section.	• Compressor power in cooling system with PCM.
• Air mass flow rate at the condenser heat exchanger inlet.	• Cooling power in normal cooling system.
• Air mass flow rate at the evaporator heat exchanger inlet.	• Cooling power in cooling system with PCM.
• Setpoint temperature of the evaporator (saturation temperature).	• COP in normal cooling system.
• The type of refrigerant.	• COP in cooling system with PCM.
• The geometric properties of condenser and evaporator.	• The temperature at different points of the cycle.
• The initial temperature of PCM.	• The amount of melting and freezing of PCM at any moment.
• The geometric properties of PCM.	• Melting fraction of PCM at any moment.
• Specific latent heat, density, and melting temperature of PCM and other thermophysical properties of PCM.	
• Charging and discharging periods of PCM.

### 3.5 Weather data

**Fig. 4:**
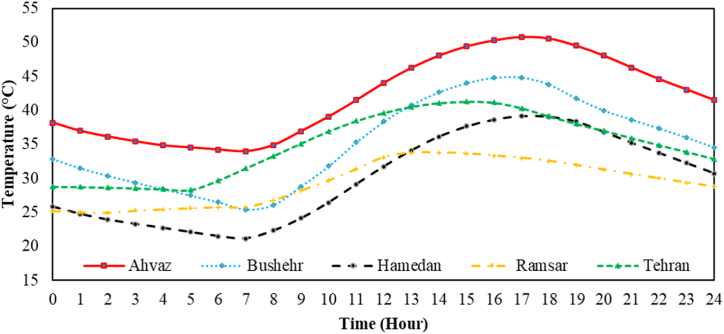
The temperature changes over 24 h for Ahvaz, Bushehr, Hamedan, Ramsar, and Tehran cities.

Five different cities named, Tehran, Ramsar, Hamedan, Ahvaz, and Bushehr have been considered for evaluating the proposed system based on various weather conditions. Their temperature changes over 24 h on their warmest days of the year are shown in Fig. 4. Ahvaz and Hamedan cities are the warmest and coldest cities among the proposed cities. The weather data have been attained from TRANSYS software and then have been imported to EES software.

## 4 Results & discussions

In this section, the results of the proposed system are presented. First, the effects of various weather conditions on the system performance are evaluated. Subsequently, the impacts of the number of PCM storage tank pipes on the system performance are analyzed. Following that, the influences of the pipe length on the system performance are investigated. It should be noted that in the base case, the number and length of pipes are considered to be 20 and 1 m, respectively.

**Fig. 5:**
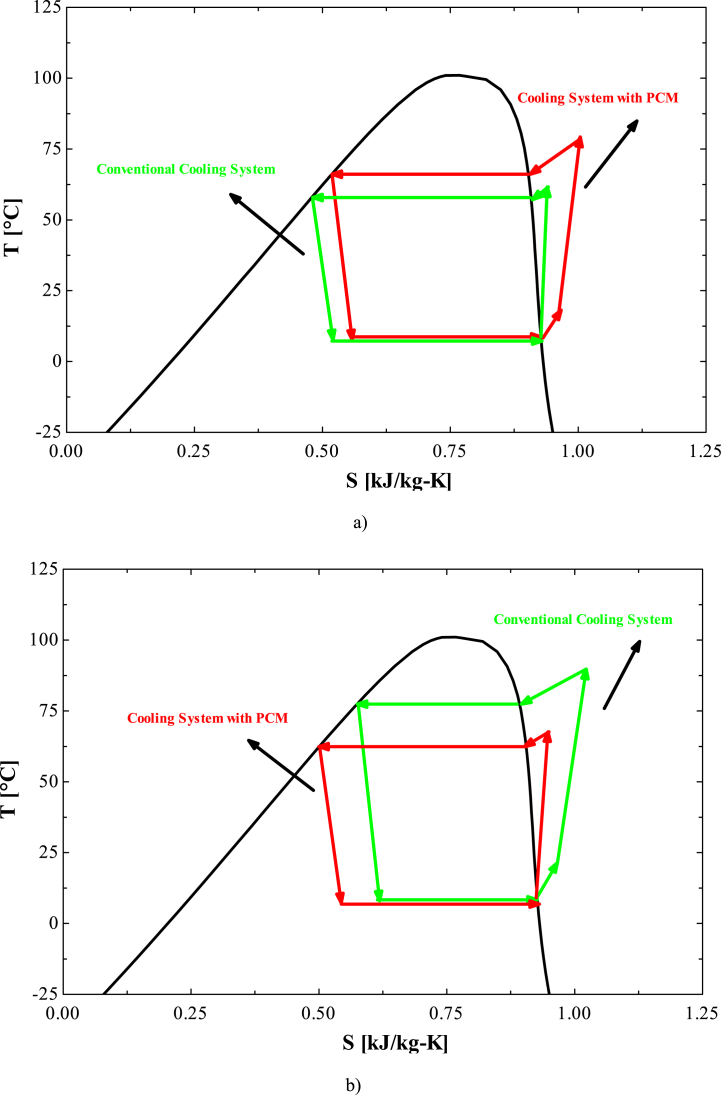
The T-S diagram of the desired vapor compression cycle a) during charging process b) during discharge process.

The T-S diagrams of the desired system at a random moment are shown in Fig. 5 for both the charging and discharging processes. As observed in the charging process (Fig. 5a), the degree of evaporator superheat is higher in the VCRS combined with the PCM storage tank compared to the conventional VCRS. This is attributed to the heat transfer between PCM and refrigerant, leading to an elevation in the evaporator outlet temperature. Consequently, the refrigerant enters the compressor at a higher temperature than in the conventional system. This, in turn, increases the temperature of the compressor outlet stream, resulting in a higher condenser saturation temperature and pressure.

During the discharge process (Fig. 5b), cooling of the condenser's inlet refrigerant stream leads to a decrease in both condenser temperature and pressure. As a consequence, the throttle valve outlet stream enters the evaporator with a lower quality compared to the conventional system. This results in a decrease in the temperature of the evaporator outlet stream, leading to a reduction in the temperature of the compressor outlet stream.

### 4.1 Various weather conditions evaluation

**Fig. 6:**
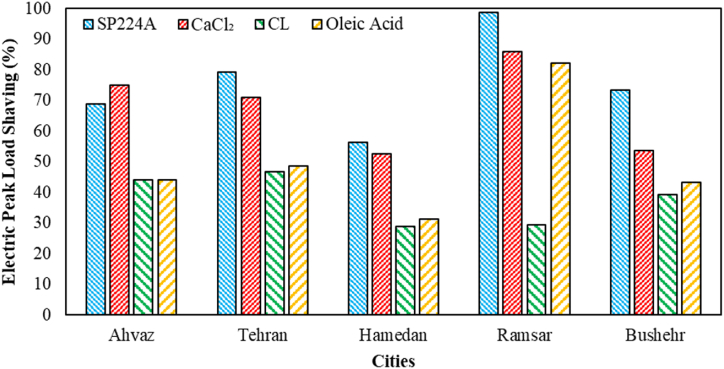
The electric peak load shaving for various PCMs based on different weather conditions.

The amounts of electric peak load shaving for various PCMs based on different weather conditions are shown in Fig. 6. As observed, SP224A has achieved the highest peak load shaving among different cities and PCMs, except in Ahvaz city. Its maximum has occurred in Ramsar city with 98.85 %. In Ahvaz city, CaCl_2_ has shown the highest electric peak load shaving with 74.96 %. In all cases, CL has had the least favorable performance, with its minimum value determined as 29.01 % based on Hamedan city weather conditions.

**Fig. 7:**
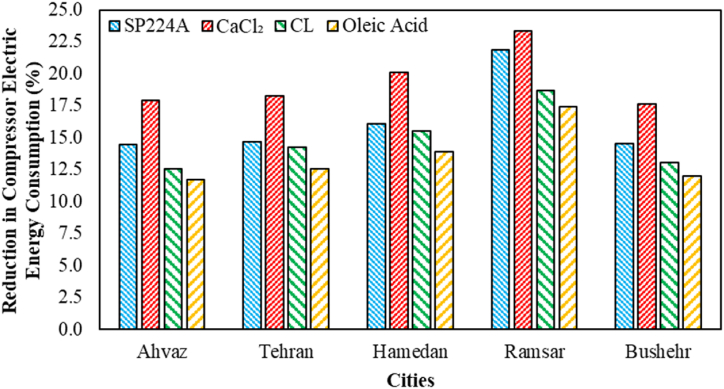
The effects of various weather conditions on the compressor's electric energy consumption during on-peak hours for different PCMs.

The effects of various weather conditions on the compressor's electric energy consumption during on-peak hours for different PCMs are indicated in Fig. 7. In all cases, CaCl_2_ has exhibited the best performance, with its maximum reduction occurring in Ramsar city at 23.38 %. Conversely, oleic acid has demonstrated the least favorable results in all cases, reaching its minimum value in Ahvaz city at 11.70 %. Notably, PCMs with higher melting temperatures have shown the maximum reduction in compressor electric energy consumption during on-peak hours in all cases. This is attributed to these PCMs storing a higher amount of cooling energy (solid PCM) during the charging process (storing cold energy in the PCM by solidifying), thanks to the increasing temperature difference between refrigerant and PCM. Meanwhile, during the discharge process (releasing cold energy from PCM by melting), PCMs with lower melting temperatures exhibit a lower temperature difference with the compressor outlet stream refrigerant, resulting in a lower heat transfer rate. Consequently, PCMs with higher melting temperatures operate for more hours during the discharge process than those with lower melting temperatures.

**Fig. 8:**
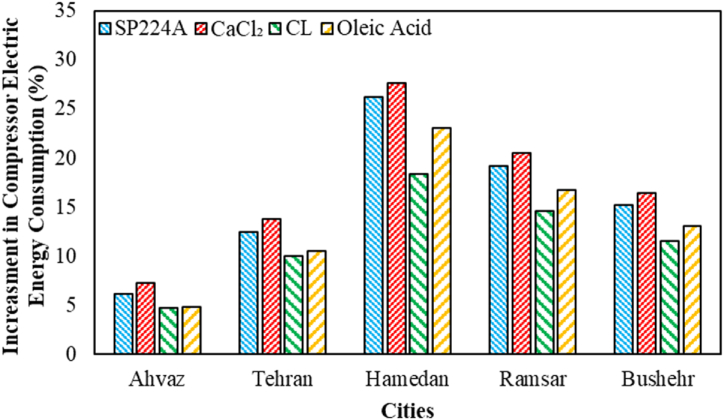
The compressor increasing electric energy consumption during the charging process (1:00 to 10:00) based on various weather conditions for different PCMs.

The compressor's increasing electric energy consumption during the charging process (1:00 to 10:00) based on various weather conditions for different PCMs is illustrated in Fig. 8. Hamedan city has shown the highest rise among various cities, reaching 27.68 %, and this is attributed to CaCl_2_. PCMs with higher melting temperatures exhibit higher electric energy consumption during the charging process (storing cold energy in the PCM by solidifying). Since the evaporator temperature is constant, PCMs with higher melting temperatures experience better heat transfer due to the increasing temperature difference between the refrigerant and PCM. Consequently, this leads to an increase in the superheat temperature value of the evaporator outlet stream.

**Fig. 9:**
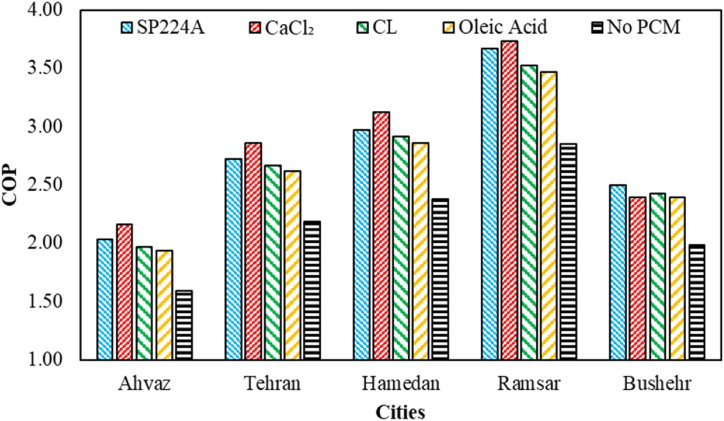
The COP of the proposed system during on-peak hours for various PCMs and based on different cities.

The COP of the proposed system during on-peak hours (12:00–19:00) for various PCMs and based on different cities is indicated in Fig. 9. As observed, CaCl_2_ has consistently achieved the best results in all cases, except in Bushehr city. The maximum COP has occurred in Ramsar city, reaching 3.74. Conversely, oleic acid has consistently demonstrated the worst performance among various PCMs. It's noteworthy that, in terms of the percentage of changes in COP compared to the base case, Ahvaz city, using CaCl_2_, has experienced the highest increase at 36.18 %. Additionally, Tehran city, utilizing oleic acid, has shown the lowest increase at 20.05 %.

**Fig. 10:**
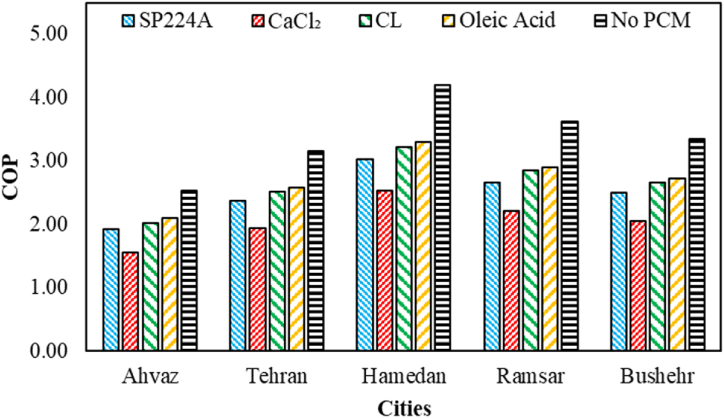
The COP of the proposed system during off-peak hours (1–10) for various PCMs and based on different cities.

The COP of the proposed system during off-peak hours (1:00–10:00) for various PCMs and based on different cities is shown in Fig. 10. Oleic acid has consistently achieved the best results in all cases, with its maximum occurring in Hamedan city at 3.30. It's noteworthy that in terms of the percentage of changes in COP during off-peak hours compared to the base case, Ahvaz city, using oleic acid, has experienced the minimum decrease in COP at 17 %. On the other hand, the highest decrease in COP during off-peak hours has occurred in Hamedan city, utilizing CaCl_2_, with 39.76 %.

### 4.2 Number of PCM pipes evaluation

In this section, the effects of the number of PCM pipes on the proposed system performance are investigated. It should be noted that the amount of refrigerant flow rate remains constant while changing the number of PCM pipes. Moreover, the results are based on Ahvaz city weather conditions.

The effects of the number of PCM storage tank pipes on the compressor's electric energy consumption and the COP of the proposed system during on-peak hours (12:00–19:00) are presented in Table 6, Table 7. In all cases, an initial increase in the number of pipes results in a reduction in the compressor's electric energy consumption, followed by an increase. The highest reduction in compressor electric energy consumption compared to the base case (17.41 kWh) is observed with CaCl_2_ using 20 pipes, amounting to about 17.97 %. The minimum reduction is associated with CL using five pipes, approximately 10.62 %

**Table 6:** The effects of the number of PCM storage tank pipes on the compressor's electric energy consumption during on-peak hours (12:00–19:00) based on Ahvaz city weather conditions.

Pipe Number	5	10	15	20	25	30
CaCl_2_ (kWh)	15.75	14.83	14.34	14.28	14.32	14.34
Oleic Acid (kWh)	16.12	15.69	15.48	15.37	15.34	15.56
SP224A (kWh)	16.01	15.43	15.02	14.89	15.04	15.27
CL (kWh)	15.56	14.94	14.80	15.21	15.53	15.80
Percentage reduction compared to without PCM mode for best case (%)	10.62	14.81	17.63	17.97	17.74	17.63

**Table 7:** The effects of the number of PCM pipes on the proposed system COP during on-peak hours (12:00–19:00) based on Ahvaz city weather conditions.

Pipe Number	5	10	15	20	25	30
CaCl_2_	1.86	2.04	2.15	2.16	2.15	2.14
Oleic Acid	1.80	1.87	1.91	1.93	1.94	1.90
SP224A	1.81	1.92	1.97	2.03	2.00	1.95
CL	1.90	2.02	2.05	1.96	1.90	1.85
Percentage increment compared to without PCM mode for best case	19.49	28.30	35.22	35.84	35.22	34.59

In all cases, increasing the number of pipes initially leads to an increase in COP during on-peak hours, followed by a decrease. The highest increase in COP is observed with CaCl_2_ using 20 pipes, reaching about 35.84 % compared to the base case. The minimum increase is associated with CL using five pipes, approximately 19.49 %.

**Fig. 11:**
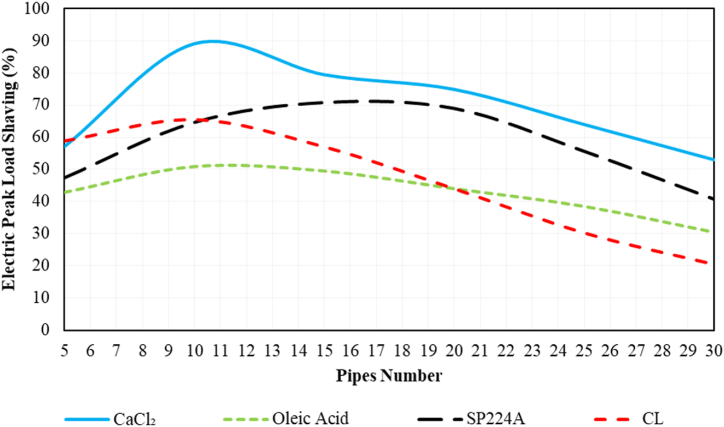
The effects of the number of PCM pipes on the electrical peak load shaving for various PCMs based on Ahvaz city weather conditions.

The effects of the number of PCM pipes on electrical peak load shaving for various PCMs are depicted in Fig. 11. In all cases, initially increasing the number of pipes leads to an increase in electric peak load shaving, followed by a decrease. The maximum electric peak load shaving, reaching 90 %, occurs with 10 pipes using CaCl_2_ as the PCM. Subsequently, SP224A achieves a peak load shaving of approximately 72 % with 17 pipes.

### 4.3 Pipes length evaluation

In this section, the effects of PCM pipe length on the performance of the proposed system have been evaluated. It should be noted that the results are based on Ahvaz city weather conditions.

**Fig. 12:**
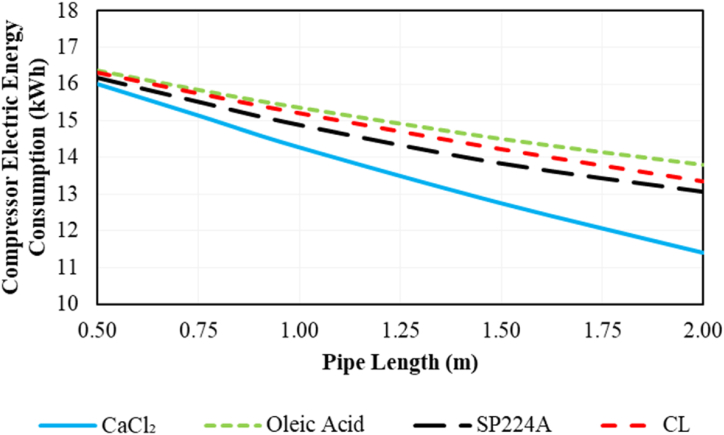
The effect of PCM pipe length on the compressor electric energy consumption during on-peak hours (12:00–19:00) based on Ahvaz city weather conditions.

The effect of PCM pipe length on compressor electric energy consumption during on-peak hours (12:00–19:00) is illustrated in Fig. 12 for various PCMs. Increasing PCM pipe length consistently leads to a reduction in compressor electric energy consumption in all cases. Among various PCMs, CaCl_2_.6H_2_O shows the highest reduction, decreasing by about 34.5 % with a pipe length of 2 m.

**Fig. 13:**
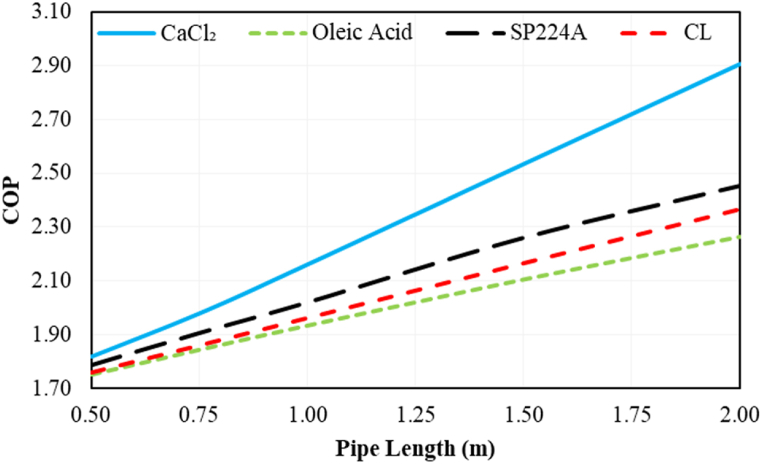
The effect of PCM pipe length on the COP of the desired system during on-peak hours (12:00–19:00) based on Ahvaz city weather conditions.

The effect of PCM pipe length on the COP of the system during on-peak hours (12:00–19:00) is illustrated in Fig. 13 for various PCMs. Increasing PCM pipe length consistently leads to a boost in COP in all cases. Among various PCMs, CaCl_2_.6H_2_O exhibits the highest COP, reaching 2.91 with a pipe length of 2 m. On the other hand, oleic acid shows the least favorable results among the various PCMs.

**Fig. 14:**
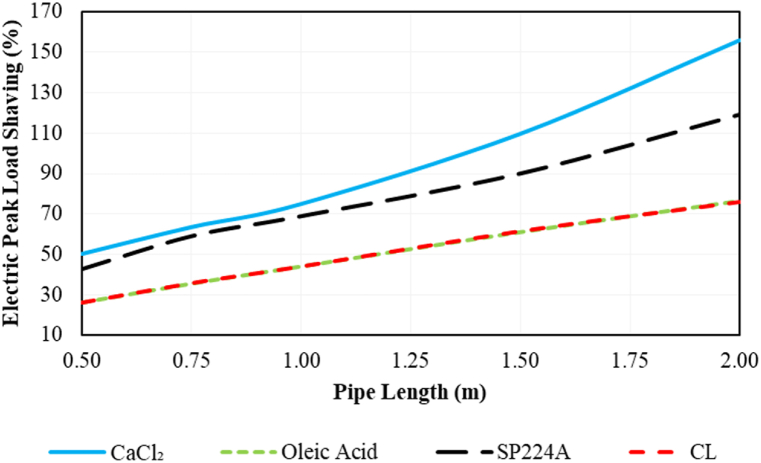
The effect of PCM pipe length on the electric peak load shaving of the desired system during on-peak hours (12:00–19:00) based on Ahvaz city weather conditions.

The effect of PCM pipe length on electric peak load shaving during on-peak hours (12:00–19:00) is indicated in Fig. 14. Increasing PCM pipe length consistently boosts electric peak load shaving in all cases. Among various PCMs, CaCl_2_ shows the best results, achieving approximately 156 % shaving with a pipe length of 2 m. This implies that the maximum compressor power consumption is lower than the average compressor electric consumption in the conventional refrigeration system.

## 5 Conclusion

In this study, a vapor compression refrigeration cycle integrated with a PCM storage tank has been simulated dynamically over 24 h. The primary objective is to reduce electric energy consumption during on-peak hours (12:00–19:00) by shifting it to off-peak hours (1:00–10:00). During off-peak hours, the vapor compression refrigeration system stores cooling energy in the PCM storage tank through the charging process. Subsequently, the stored cooling energy is utilized during on-peak hours to pre-cool the condenser inlet refrigerant in the discharging process. This approach enhances the cooling system's performance and reduces electric consumption during on-peak hours. The selected PCM media include oleic acid, CL, SP224A, and CaCl_2_.6H_2_O. The study investigates the effects of weather conditions and PCM storage tank parameters on the system's performance. Key results are presented below.

• Among various PCMs evaluated in different cities, SP224A achieved the highest electric peak load shaving, except in Ahvaz city. Its maximum peak load shaving occurred in Ramsar city, reaching 98.85 %.
• In terms of compressor electric energy consumption during on-peak hours (12:00–19:00), CaCl_2_ consistently exhibited the lowest consumption in all cases, with the maximum reduction occurring in Ramsar city at 23.38 %.
• The sensitivity analysis on the number of PCM storage tank pipes indicates that as the number increases, electrical peak load shaving initially rises to its maximum value before gradually decreasing.
• The sensitivity analysis on the length of PCM pipes reveals that, in all cases, increasing pipe length results in a reduction of compressor electric energy consumption during on-peak hours (12:00–19:00). Additionally, it leads to an increase in COP during the specified time.
• By increasing the length of PCM storage tank pipes, electrical peak load shaving has improved in all cases. The best results are achieved using CaCl_2_ as the PCM medium with a pipe length of 2 m, leading to electrical peak load shaving of about 156 %.

## Data availability statement

The data generated during this study are included in the manuscript information.

## CRediT authorship contribution statement

**Alireza Riahi:** Writing – review & editing, Writing – original draft, Investigation. **Mohammad Behshad Shafii:** Writing – review & editing, Supervision, Conceptualization.

## Declaration of competing interest

The authors declare that they have no known competing financial interests or personal relationships that could have appeared to influence the work reported in this paper.

## Figures and Tables

**Table:** 

Abbreviation	
COP	Coefficient of Performance
CL	Eutectic Mixtures Of 45 % Capric Acid And 55 % Lauric Acid by Weight
EES	Engineer Equation Solver
IEA	International Energy Agency
NTU	Number of Transfer Unit
PCM	Phase Change Material
SP224A	Commercial Blend of Salt Hydrate and Paraffin Wax
VCRS	Vapor Compression Refrigeration System
Symbols	
h	Enthalpy (kJ/kg. K)
$\dot{m}$	Mass Flow Rate (kg/s)
P	Pressure (kPa)
${\dot{Q}}_{evap}$	Evaporator Cooling Load (kW)
R	Thermal Resistance (m^2^. K/W)
r	Radius (m)
T	Temperature (°C)
$V_{G}$	Compressor Inlet Volume (m^3^)
${\dot{W}}_{comp}$	Compressor Power Consumption (kW)
Subscript	
5	Compressor Inlet
6	Compressor Outlet
C	Cold fluid flow
Comp	Compressor
cond	Condenser
elec	Electrical
Evap	Evaporator
in, i	Inlet
is, isen	Isentropic
mech	Mechanical
o, out	Outlet
Greek Letter	
$\eta_{is}$	Isentropic efficiency
$\eta_{v}$	Volumetric efficiency
ε	Heat exchanger effectiveness
ρ	Density (kg/m^3^)
ω	Compressor speed (rpm)
